# Insights from search summary tables for evidence and gap maps: a case study on peer support interventions

**DOI:** 10.5195/jmla.2025.1831

**Published:** 2025-04-18

**Authors:** Alison C. Bethel, Naomi Shaw, Rebecca Abbot, Morwenna Rogers, Anna Price, Rob Anderson, Sian de Bell, Jo Thompson Coon

**Affiliations:** 1 a.bethel@exeter.ac.uk, NIHR ARC South West Peninsula (PenARC), University of Exeter Medical School, University of Exeter, Exeter, United Kingdom; 2 n.shaw2@exeter.ac.uk, HS&DR, University of Exeter Medical School, University of Exeter, United Kingdom; 3 r.a.abbott@exeter.ac.uk, NIHR ARC South West Peninsula (PenARC), University of Exeter Medical School, University of Exeter, Exeter, United Kingdom; 4 morwenna.rogers@exeter.ac.uk, NIHR ARC South West Peninsula (PenARC), University of Exeter Medical School, University of Exeter, Exeter, United Kingdom; 5 a.price@exeter.ac.uk, University of Exeter Medical School University of Exeter, Exeter, United Kingdom; 6 r.anderson@exeter.ac.uk, ESMI, University of Exeter Medical School University of Exeter, Exeter, United Kingdom; 7 s.c.de-bell@exeter.ac.uk, HSDR Evidence Synthesis Centre, University of Exeter Medical School, University of Exeter, Exeter, United Kingdom; 8 J.Thompson-Coon@exeter.ac.uk, NIHR ARC South West Peninsula (PenARC), University of Exeter Medical School, University of Exeter, Exeter, United Kingdom

**Keywords:** Evidence synthesis, evidence and gap maps, information retrieval, search summary table

## Abstract

**Background:**

Evidence and Gap Maps (EGMs) are a visual representation of the available evidence relevant to a specific research question or topic area. They are produced using similar methods to systematic reviews, however, there is little guidance on which databases to search and how many. Information Specialists need to make decisions on which resources to search, often for a range of study designs within a broad topic area to ensure comprehensiveness.

**Case Presentation:**

This case study presents two search summary tables (SSTs) from an evidence and gap map on peer support interventions. The first search summary table presents the findings of the search for systematic reviews and the second for randomised controlled trials. Different databases and different searches were undertaken for the two different study types.

**Conclusion:**

The two SSTs indicated that MEDLINE and PsycINFO were key databases required for the identification of both systematic reviews and randomised controlled trials of peer support interventions, with the addition of CINAHL for systematic reviews, and CENTRAL for randomised controlled trials. For both study types, forward citation searching found additional included studies although it was more lucrative for identifying additional randomised controlled trials. Search summary tables are a simple way to share the effectiveness of the search methods chosen for a specific evidence synthesis project. The more SSTs we have, the more data we will have to inform evidence-based decisions on our search methods.

## BACKGROUND

Evidence and Gap Maps (EGMs) are a visual representation of the available evidence relevant to a specific research question or topic area. They facilitate evidence-informed decision-making in many areas including health, social care, education, and environmental science, by creating an accessible overview of existing systematic reviews and/or primary research on a given topic [1]. While they provide an overview and categorise/group relevant studies within the same format, unlike other forms of systematic review they do not seek to synthesise the evidence across studies. EGMs can also highlight where there is a lack of evidence, indicating areas for investment in future primary research and evidence synthesis [2].

EGMs are produced using similar methods to systematic reviews, following guidance to ensure the conduct and reporting of each step is transparent, robust, and reproducible. Snilstveit et al. (2016) [1] outline the main steps of an EGM: determining the scope and framework for the EGM in consultation with key stakeholders, developing study eligibility criteria, systematically searching for relevant studies, screening studies against eligibility criteria, data extraction providing summaries and appraisals of included studies, and finally creating a visual display of studies. While sharing similar objectives to other “big picture” review types, EGMs are proposed to differ from scoping reviews in several ways. EGMs may take an even ‘broader’ perspective to a topic area than scoping reviews, typically including a greater number of studies, with higher level data extraction and employing visual, interactive outputs to represent findings. Through use of a structured, pre-specified framework to guide categorisation and mapping of studies, EGMs provide a systematic approach to the identification and presentation of gaps in the literature [3-5].

Existing guidance on methods for EGMs (Table 1) does not provide a clear indication of the number of sources, or requirements for key databases to be searched to identify studies. For example, The Campbell Collaboration guidance for the conduct of searches for EGMs [6] requires searches to be “systematic and cover a broad range of literature” and to “ensure that the search includes appropriate national, region, and subject specific bibliographic databases”, while the Collaboration for Environmental Evidence [7] suggests starting the search by targeting sources likely to retrieve the largest number of relevant references. Furthermore, no specific guidance for the conduct of EGMs for clinical healthcare topics was identified.

**Table 1:** Guidance on search methods for Evidence and Gap Maps (EGMs)

Organisation	Guidance
Campbell EGM conduct standards [6]	Ensure that the search includes appropriate national, regional, and subject specific bibliographic databases. Searches should be systematic and cover a broad range of literature, keeping in mind that they cannot always be as comprehensive as a systematic review because of the broad scope. Ensure the search strategy is sufficiently broad to not miss any bodies of literature. There is no minimum set of databases to search, but authors should consider consulting with a research retrieval specialist to avoid unnecessary duplication of effort.
Collaboration for Environmental Evidence [7]	“…the review team should start the search using the source where the largest number of relevant papers are likely to be found, and subsequent searches can be constructed with the aim to complement these first results.”
Snilstveit, 2016 [1]	“The search strategy should be systematic and comprehensive but also needs to be manageable. One of the benefits of EGMs is that they can be done relatively quickly, so there is a need to strike a balance between an exhaustive and feasible search.” “Other techniques that can increase the efficiency of the search include focusing on key repositories of impact and systematic reviews. Authors should supplement searches with subject specific searches in academic databases and relevant web sites. Other techniques such as snowballing citation tracking, and use of listservs. Finally, text-mining…. reducing the time and workload of identifying studies for inclusion.”

For EGMs, information specialists are faced with the challenge of designing and conducting systematic searches, often for a variety of study designs in broad topic areas, while ensuring this is manageable within the time available. Due to the broad topic areas more databases may need to be searched, and the terminology required may lead to increased search results, therefore, information specialists need to carefully consider their approach, prioritizing searches of key sources and using search techniques that will ensure comprehensiveness, while avoiding duplication of effort in searching sources that are unlikely to yield additional relevant records.

Reporting standards for EGMs [6, 8] require description of all sources and full search strategies for each database to be reported. However, there is currently no requirement to report details of search effectiveness or provide an evaluation of database selection or different search approaches used. The reporting guidelines for searching, PRISMA-S [9], requires the information sources and methods used, search strategies, peer review and managing records to be reported but does not include any evaluation or effectiveness of the search. SearchRxiv (https://www.cabidigitallibrary.org/journal/searchrxiv) was set up in 2022 as a place for anyone to deposit their searches in any discipline, again, no evaluation is required. Search summary tables (SST) [10] offer a simple way to provide an overview of the results of the searches in all types of evidence syntheses, indicating which database searches found included references and which supplementary search methods found additional unique references, as well as sensitivity and precision calculations for each database. SSTs have been completed for systematic reviews [11] and provide useful insights for identifying optimal approaches in searches for guideline development [12] and programme theories [13].

Publishing SSTs [10] for an EGM, alongside their search methods and the PRISMA flow diagram [9], adds to the growing evidence base about the contribution of individual databases and supplementary search methods for study identification. They can also inform efficient use of sources and searches for any planned updates to the EGM. Furthermore, creating SSTs for EGMs that include multiple study designs may provide insights into the usefulness of different sources and approaches for identifying a variety of study designs (e.g., systematic reviews, randomised controlled trials and economic evaluations).

The aim of this study was to develop and explore the utility of SSTs for different types of included studies: systematic reviews (SR), randomised controlled trials (RCT) and economics evaluations (EE), using a case study of an EGM on peer support interventions.

## CASE PRESENTATION

The first two authors conducted database searches and supplementary searches for an EGM on peer support interventions [14].

All records retrieved from searches including duplicates, were downloaded into EndNote X9.2 (Clarivate) and coded to indicate the source (i.e., name of bibliographic database or supplementary search method). The number of records retrieved from each database, those screened at Title/Abstract stage (after deduplication), and those screened at Full-Text were also documented.

Search summary tables (SSTs) were created in Excel using a template and process developed by Bethel [10] involving two phases. The first phase of the SST creation involved adding details of included references to separate SSTs for each study design (systematic reviews, randomised controlled trials, and economic evaluations). The data in the SST for EEs were incomplete so it has not been included in this case study.

The second phase of the SST creation involved searching for each included reference in selected bibliographic databases (for SRs: CINAHL, MEDLINE, PsycINFO, Embase and Epistemonikos; for RCTs: MEDLINE, CENTRAL, CINAHL and PsycINFO; using article titles or accession numbers), and re-running searches to determine if an updated search using the original search strategy would retrieve included references not located by the original search. This information was also recorded in the SST.

Two SSTs were produced, one for systematic reviews (SRs) and one for randomised controlled trials (RCTs). Tables 2 and 3 provide a summary of the SST findings for both study designs. The full SSTs are available in the Supporting Information published with Price [13, 14].

**Table 2:** Summary findings from an SST for Systematic Reviews included in EGM

Source	Refs retrieved	Included refs (%)	Unique included refs from original search (October 2020)	Additional refs in search re-run (January 2021)	Refs in database not retrieved by search
Total included references	N/A	32 (100%)	N/A	N/A	N/A
Cochrane Database of Systematic Reviews	52	2 (6%)	0	N/A	N/A
CINAHL	877	23 (72%)	2	2	0
MEDLINE	1123	27 (84%)	2	2	1
PsycINFO	581	15 (47%)	1	3	1
Embase	1484	25 (78%)	0	2	1
ProQuest Dissertations & Theses Global (PQDT)	807	0	0	N/A	N/A
Applied Social Science Index and Abstracts (ASSIA)	188	4 (12%)	0	N/A	N/A
Epistemonikos	332	7 (22%)	0	5	20
Forwards Citation Searching (FCS)		1 (3%)	1		
Backwards Citation Searching (BCS)		0	0		
Google Scholar		1 (3%)	1		

**Table 3:** Summary findings for an SST for Randomised Controlled Trials included in EGM

Source	Refs retrieved	Included refs (%)	Unique included refs from original search (March 2021)	Additional refs in search re-run (September 2021)	Refs in database not retrieved by search
Total included references	N/A	61 (100%)	N/A	N/A	N/A
MEDLINE	3190	50 (82%)	1	4	4
CENTRAL	4836	46 (75%)	1	7	4
CINAHL	2026	37 (61%)	0	8	1
PsycINFO	224	25 (41%)	2	2	1
FCS (all sources)		7 (11%)	4		
BCS		0	0		
Google Scholar		5 (8%)	1		

The SR searches included eight bibliographic databases and three methods of supplementary searching (forward and backward citation searching of included references identified from database searches, plus Google Scholar searches). The SR SST indicates that of these searches, only three bibliographic databases and two supplementary search methods retrieved unique references. Unique references are those references retrieved by a database search that were not retrieved by any other database search. As indicated in Table 2, forward citation searching found the additional included article [15] which was identified by all three sources used (Web of Science, Scopus and CitationChaser). Backward citation searching found no further additional relevant SRs.

For the second phase of the SST, CINAHL, MEDLINE, PsycINFO and Epistemonikos were searched to determine which of the 32 included SRs were available in each database, and whether these references would be retrieved in update searches using the original search strategies. The original MEDLINE searches (completed October 2020) did not retrieve five of the 32 included references. However, when the searches were re-run for the SST analysis in January 2021, two articles [15, 16] identified through supplementary searches, were found to be in MEDLINE. An additional reference [17] was indexed within MEDLINE but was not retrieved by our original search strategy. Two further included references [18, 19] were not in MEDLINE at all.

Epistemonikos was found to include all 32 SRs; however, the SR SST indicated that our search strategy did not retrieve 20 of them. This prompted us to investigate issues with the search strategy and check our understanding of search functionality within Epistemonikos.

The RCT searches included four bibliographic databases (CENTRAL, CINAHL, MEDLINE and PsycINFO) and three supplementary search methods (forward and backward citation searching of the included references identified from the database searches, plus Google Scholar searches). Table 3 provides the summary findings and Appendix A provides further details of the outcomes of forward citation searching using different tools.

When the database searches were re-run for the second phase of the SST analysis in September 2021, five of the references not retrieved by the original bibliographic database searches (completed in March 2021) were found. Four were in MEDLINE and one in PsycINFO. As seen in Table 3, four references [20-23] were not found by the search strategies used in those databases but were available in MEDLINE. Three references used terms to describe peer support interventions (i.e., lay tutors, lay volunteers and navigators) that had not been included in search strategy, and one reference [20] did not include any terms from the RCT filter. Only one RCT [24] was not available in the any of the bibliographic databases searched for this review. This RCT [24] is a PhD thesis retrieved from Google Scholar searches.

## DISCUSSION

The SSTs confirm that there is value in searching multiple bibliographic databases to identify SRs and RCTs relevant to peer support interventions due to differences between databases in subject coverage, available publication types and the application of controlled vocabulary terms. Furthermore, the SSTs for this EGM provide data on the key databases to prioritise for search updates for SRs and RCTs, with a limited selection potentially minimising the number of records needed to screen to identify included studies.

Analysis of SSTs indicated that MEDLINE and PsycINFO were key databases required for the identification of both SRs and RCTs of peer support interventions, with the addition of CINAHL for SRs, and CENTRAL for RCTs. Our findings are consistent with research and guidance on database selection for evidence syntheses within health and social care topic areas that points to MEDLINE as a key source [25-27], but also that searches of multiple bibliographic databases are essential for comprehensive retrieval of relevant studies [28-30]. This case study also indicates that SST-informed database selection could minimise the number needed to screen for search updates. For example, a search approach for SRs prioritising three databases (MEDLINE, PsycINFO and CINAHL) would retrieve 2,581 records, compared with 5,444 records for a search of eight databases.

The SST also provided the data for us to further investigate the search terms and controlled vocabulary terms used in the bibliographic database searches. It provided evidence for what we might do in the future for both the subject specific terms and study filter terms.

Epistemonikos (epistemonikos.org) has been highlighted as a potentially useful resource for identifying systematic reviews on health-related topics, with Goosen [25] suggesting a combination of MEDLINE and Epistemonikos searches alongside reference list checks as the optimal approach. However, this was determined by checking source coverage (by searching to see if the SR was contained in the database), rather than through application of search strategies to retrieve a set of known SRs. Reviewing the SST for SRs in this case study indicated a potential issue with our search strategy for Epistemonikos. We noted that while all 32 (100%) of the included references were available in Epistemonikos, our original search strategy only retrieved 7 (22%) of these. We were able to pinpoint an issue with searching for hyphenated phrases (e.g., peer-support) in Epistemonikos. For updates for this EGM, and in future searches, we intend to search Epistemonikos with a simplified strategy. We also recommend that Epistemonikos provide further guidance on advanced search functionality to support effective searching for evidence syntheses.

Guidance for searching for EGMs recommends use of supplementary methods such as searches of relevant websites and citation searching [1]. Research has demonstrated the value of citation searching for the identification of additional studies for evidence syntheses, particularly in topic areas where there is vague or inconsistent terminology [31]. The TARCiS statement [32] recommends that further research is needed in this area, and this case study highlights how SSTs can support this research priority, with the SST clearly indicating the methods of citation searching undertaken, and whether these added any value. For this EGM, forward citation searching added unique results for the RCTs, but not for the SRs, when compared with updating the searches prior to project completion. Backward citation searching did not yield any unique included SRs or RCTs. However, the eligibility criteria for this EGM excluded studies published prior to 2015. It is possible that backward citation searching may have yielded relevant studies if eligibility criteria included older publications.

This study also adds to existing research investigating the comparative usefulness of different tools for citation searching [33-35], thus supporting the third research priority in the TARCiS statement *“Further research is needed to assess the best way to perform citation searching,”* and second statement *“optimal use of indexes and tools and their combination to conduct citation searching”*. The SSTs showed duplication in the references retrieved in forward citation searching by Scopus, Web of Science and Citation Chaser [41]; however, Scopus identified the most references overall for both RCTs and SRs. This could be topic dependent, although Rogers [35] also found forward citation searching with Scopus yielded a greater number of included studies than Web of Science in a review of implementation studies on dementia care. For EGMs, there is a need to strike a balance between comprehensiveness and an effective use of time and resources. Through reporting which tools were used and their effectiveness, information specialists could make evidence-informed decisions on the most appropriate tool, and whether citation searching of multiple indexes can be justified in the time available. Forward and backward citation searching on Scopus and Web of Science currently involves searching reference by reference to retrieve and download cited and citing references. Workarounds using EndNote have been proposed for Scopus and Web of Science [36], however, free specific tools such as CitationChaser provide functionality for the bulk upload of digital object identifiers (DOIs) or other unique identifiers (e.g., PMIDs, PMCIDs) allowing for concurrent citation searching on multiple references. Furthermore, CitationChaser [37] is a freely available tool, whereas both Web of Science and Scopus are subscription services.

The Campbell Collaboration standards for the conduct of EGMs include grey literature searches as a mandatory requirement [6]. While searching for grey literature can help minimise publication bias [33], the Collaboration for Environmental Evidence [7] notes the time-consuming nature of these searches. In this EGM, we conducted searches of Google Scholar to identify additional SRs and RCTs, however, we searched ProQuest Dissertations & Theses for SRs only. Google Scholar searches yielded one included RCT, a PhD thesis, so this has prompted us to reconsider searching ProQuest Dissertations & Theses for RCTs, either as an alternative to Google Scholar or as an additional source.

Publication of SSTs alongside detailed search methods for EGMs and other evidence syntheses would ensure processes for the identification of studies are fully transparent. Currently, the main drawback would be the cost associated with the time taken to do this; however, tools are in development that may facilitate SST creation. Sharing SSTs would enable information specialists and other researchers to make evidence-based decisions regarding appropriate database selection and search techniques when working on a similar topic or research question. This may be particularly important for EGMs where exhaustive searching is not feasible or appropriate. SSTs could help researchers and information specialists target key databases to search as a minimum, or to prioritise the most fruitful supplementary search methods. This may, in turn, minimise the number of records needed to screen. They could help prioritise which databases to begin the search in, as recommended by some of the current guidance [7]. Use of SSTs could also help streamline processes to identify new studies for inclusion in ‘living’ EGMs.

The SSTs for this EGM on peer support interventions demonstrated that for SRs, a search of multiple bibliographic databases, plus either supplementary searching, or a search update of MEDLINE were required to comprehensively identify all included references. A combination of database and supplementary searches were necessary to identify all included RCTs. This was a single case study of one EGM for one topic area (peer support interventions) for two study designs (systematic reviews and randomized controlled trials), and it may be difficult to make generalisations from these SSTs to other research questions or topic areas. The EGM also included economic evaluations; however, we do not have the detailed data for this to provide sufficient information for discussion.

This case study demonstrates that new insights can be gained from completing SSTs at the end of an evidence synthesis project. Reporting a search summary table (SST) alongside full search strategies would be a useful addition to any evidence synthesis publication, either in supplementary materials, or accessible through institutional repositories or collections of search strategies (e.g. SearchRxiv). Over time, SSTs could be considered for inclusion in future iterations of the PRISMA-S guidance. This would allow full transparency of search processes, support reflective practice, add to our existing literature on evidence-based searching, and provide opportunities for future research to improve the efficiency of search methods for EGMs and other types of evidence synthesis. Ideally, a technical solution could be developed to for populating a SST. The more SSTs we have, the more data we will have to inform evidence-based decisions on our search methods.
