# Supplementary material for: Insights from search summary tables for evidence and gap maps: a case study on peer support interventions
Source: J Med Libr Assoc. 2025 Apr 18;113(2):177–83. doi: 10.5195/jmla.2025.1831 (PMC12058345; doi:10.5195/jmla.2025.1831)
Supplement: Supplementary file 1 — Appendix A [file jmla-113-2-177-s01.docx]

Appendix A

| ***Forward citation searching: Sources of additional RCTs*** | | | | |
| --- | --- | --- | --- | --- |
| **Included references** | **Format** | **Supplementary searches** | | |
|  |  | ***fcs scopus*** | ***fcs wos*** | ***fcs cit chaser*** |
| Andreae, 2021, Peer coach | jnl | *x* |  |  |
| Andreae, 2021, Peer delivered, linked | jnl |  |  | x |
| Hundert, 2021, linked | jnl | x |  |  |
| Kidd, 2021 | jnl | x | x | x |
| Kyaw Tha Tun, 2021 | jnl | x |  |  |
| Larsen, 2019 | jnl | x | x |  |
| Sampson, 2021 | jnl | x | x | x |
| Sullivan, 2018 | jnl | x | x | x |
| No. included refs |  | 7 | 4 | 4 |
| No. unique refs |  | 2 | 0 | 1 |
